# Psychometric properties of the metacognition questionnaire (MCQ-A) in Spanish adolescents

**DOI:** 10.1186/s41155-025-00347-0

**Published:** 2025-04-22

**Authors:** Carlos Salavera, José L. Antoñanzas, Eva Urbón, Pablo Usán

**Affiliations:** 1 Investigation Research Group OPIICS, Universidad de Zaragoza, Saragossa, Spain; 2 Cátedra TEA Ediciones Universidad de Zaragoza, Saragossa, Spain; 3 Universidad de Zaragoza, c/Pedro Cerbuna, 12, Saragossa, 50009 Spain

**Keywords:** Metacognition, Adolescents, MCQ-a, Factorial Structural, Network analysis

## Abstract

**Background:**

The Metacognition Questionnaire, including the version adapted to adolescents, measures non-adaptive metacognition believes. This study measured the structure of the MCQ-A factor in adolescents, and its correlation with anxiety and emotional regulation.

**Objective:**

The aim of this study was the adaptation and validation of the Spanish version of the questionnaire on metacognitive beliefs in adolescents with a sample of schoolchildren (*N* = 1031, age = 14.91 years).

**Methods:**

Two studies were undertaken: (1) the translation of the MCQ-A scale into Spanish, including the evaluation of internal consistency, factorial structure, and convergent validity; and (2) the confirmatory factorial analysis of the questionnaire.

**Results:**

Five factors were obtained: (1) positive beliefs about concerns; (2) negative beliefs about lack of control and concern risk; (3) cognitive confidence; (4) the need to control thoughts; and (5) cognitive self-awareness. The exploratory factorial analysis clearly showed that the MCQ-A scale factors present an aggregate variance of 53.42%, which explains the unique variation of metacognitive beliefs. For its part, confirmatory factorial analysis endorsed the suitability of the model, with a sustainable structure that comprise the five factors identified and 30 items. In addition, network analysis revealed that metacognitive beliefs and feature-anxiety are related.

**Conclusion:**

The MCQ-A is easy to understand and fast to complete, so it is regarded as useful for the assessment of non-adaptive metacognitive beliefs in adolescents.

## Introduction

Metacognition refers to the knowledge and regulation of one's own cognitive processes, that is, the ability to monitor, control, and plan activities related to learning and problem-solving. This involves both knowledge about how one learns and the ability to adjust cognitive strategies to improve the individual’s understanding and performance (Brown, 1987; Flavell, 1979; Schraw & Dennison, 1994; Winne & Azevedo, 2014; Zhang, 2021).

The study of metacognition has been shown to be a key learning strategy (Frith, 2023a, 2023b; Heyes et al., 2020), and learning strategies have become one of the most important tools in the acquisition of knowledge. Muñoz (2005) points out that learning strategies comprise thoughts, behaviours, beliefs, and emotions, which facilitate the acquisition of information and link it with previous knowledge (Broadbent & Poon, 2015; Weinstein, 1987; Weinstein et al., 2000). Learning strategies can also be described as a way to learn to learn (Novak & Gowin, 1988; Pozo et al., 2001), in allusion to thought’s metacognitive capabilities.

Therefore, it can he argued that learning strategies are integrated sequences of actions or activities that are deliberately chosen in order to facilitate the acquisition, storage, and use of information (Anthonysamy et al., 2020).

Metacognition is, in general, defined as the awareness of personal knowledge. For Flavell (1976), it is the individual’s knowledge about their own cognitive processes and products, and everything related to them. Metacognition is, among other things, and active form of supervision that regulates and organises said processes in relation to specific cognitive goals (Schraw et al., 2006). This knowledge can refer to tasks, persons, or their strategies. Crespo (2004) defines the three types of knowledge as follows: (1) task knowledge refers to an individual’s perception of the influence that the nature of a task has upon its fulfilment. This can be related to the type of information encountered in a given cognitive task. Memorising a phone number and a twenty-item shopping list are very different things. This type of knowledge is more directly related to the type of demand posed by a cognitive task. When the subject knows that they are taking a physics lesson, they will find it easier to remember the elements; (2) Knowledge about persons relates to what the subject knows about human beings as cognitive agents. This can be linked to the notion of existence, distinction, and integration, as defined in Wellman’s (1985) “theory of mind”. However, Flavell (1993) does not linger to analyse these elements in such detail, but focuses on who the knowledge refers to, and divides it into three categories. The first refers to what a person knows or thinks about their cognitive abilities and shortcomings. It is an intraindividual belief emerging from the individual’s accumulated experience as a cognitive agent. The second refers to differences perceived between the subject’s skills and those of others. It is the intersubjective belief that emerges from the observation of our interaction with other subjects. Finally, knowledge about cognitive abilities shared by all people. A subject will develop an approximate understanding of the memory skills of their interlocutors after providing information, in the knowledge that passing time will affect it. Flavell (1993) defines it as an understanding of the universal properties of cognition, especially in normal conditions. As such, it is considered the most interesting subcategory of knowledge, and its everyday importance is stressed; (3) Finally, knowledge of strategies refers to knowledge of the differential value of cognitive and metacognitive strategies to meet a given goal.

Metacognitive knowledge comprises a person’s knowledge of human, and their own, thought processes; the latter is related to the knowledge that subjects have of their own personal and idiosyncratic cognitive resources (Shea & Frith, 2019).

Summing up, most models divide metacognitive activity into two processes: (a) understanding of human cognition; and (b) ability to handle cognitive resources, that is, knowledge about cognitive processes and their regulation (Martí, 1999).

As noted by Brown (1981), the two components of metacognition, knowledge and regulation, are conceptually different but are related, which is important for a global and more comprehensive idea of metacognition.

Therefore, regulation improves the use of cognoscitive resources, such as attention, strategy, and awareness of understanding difficulties. Different studies emphasise that subjects that have been trained in regulation significantly improve their learning skills (Barahmand, 2009; Robson et al., 2020; Wennerhold & Friese, 2022).

On the other hand, Brown (1987) pointed out that regulation, planning, supervising, and evaluating can be unconscious and impossible to outline in some learning settings. Many of these processes are automatic, at least in adults; in addition, many of them have developed without conscious reflection, and are, therefore, difficult to self-report.

To measure metacognition in adolescents, there are different instruments: the Metacognitive Awareness Inventory (Schraw & Dennison, 1995); the Learning Self-Regulation Scale (Pintrich, 1991); the Metacognitive Strategies Questionnaire (Veenman et al., 2006); the Metacognitive Awareness Questionnaire for Adolescents (Kaya et al., 2015); and the MCQ-A Metacognitive Questionnaire (Wells & Cartwright-Hatton, 2004), among others.

For this study, the MCQ-A Metacognitive Questionnaire for Adolescents (Wells & Cartwright-Hatton, 2004) was selected because it is a tool specifically designed to assess metacognition in adolescents. Widely used in both educational settings and research, it measures metacognitive awareness and self-regulation strategies in adolescents, allowing for the assessment of their ability to monitor, control, and regulate their cognitive processes. Some advantages of the MCQ-A include its multidimensional assessment of metacognition (metacognitive knowledge and metacognitive regulation), its facilitation of self-reflection, and its ability to identify areas for improvement, making it a useful tool for educational interventions. Additionally, it is easy to administer and apply, and it is aligned with modern theories of metacognition and self-regulation, such as Flavell’s metacognitive model and Zimmerman’s self-regulation model.

The questionnaire presents a stable five-factor structure: (1) metacognitive knowledge (the awareness adolescents have of their own cognitive processes); (2) planning (the ability of adolescents to anticipate tasks and organise themselves before starting work); (3) monitoring (how adolescents monitor their progress and understand how they are advancing during the learning process); (4) evaluation (the ability of adolescents to reflect on their own performance after completing a task); and (5) cognitive regulation (the ability to control and adjust cognitive processes during the task). In summary, the MCQ-A measures how adolescents know, plan, monitor, evaluate, and regulate their cognitive processes, which is essential for promoting autonomous and effective learning.

The structure of the MCQ has been replicated in scales adapted for epilepsy (Fisher et al., 2016), obsessive–compulsive disorder (Grøtte et al., 2016), and breast cancer (Cook et al., 2014). To our knowledge, the MCQ-A has not been previously applied to Spanish adolescents.

This study has two main objectives: (1) to examine the MCQ-A’s five-factor structure; and (2) to examine the concurrent validity of MCQ-A and anxiety and emotional regulation values.

The only hypothesis is that the MCQ-A is a valid instrument to measure metacognition in adolescents.

## Method

### Participants

The sample comprised 1031 secondary school students: 509 males (49.37%) and 522 females (50.63%); the average age of participants was 14.91 years, ranging from 12 to 17 years (standard deviation 1.61). The first study comprised 525 participants (258 males and 267 females), and the second 506 participants (251 males and 255 females). The educational centers were divided between public (*N* = 614; 59.55%) and private schools (*N* = 417; 40.45%). Additionally, a 73.42% of the participants (*N* = 757) belonged to the urban environment and a 26.58% (*N* = 274) to the rural environment. All participants took part in the study voluntarily and signed an informed consent form, following all the guidelines of the Declaration of Helsinki and all ethical criterial for research with human beings (informed consent, right to information, protection of personal data and confidentiality, non-discrimination, gratuity, and the option to abandon the study at any point). In order to ensure that the questionnaires were being correctly administered, a small group of participants (*n* = 56) was approached before the study to confirm that all items were correctly understood. The questionnaires were handed over in the classrooms in the presence of the principal investigator. The questionnaires were collected individually as they were completed, so that they could be checked for unanswered items. The study was found to be representative (Botella et al., 2012) of the province of Zaragoza, with a confidence level of 99% and a sampling error of 5%.

### Instruments

#### MCQ-A questionnaire (Wells & Cartwright-Hatton, 2004)

This 30-item scale is based on the MCQ- 30. The language has been simplified slightly to ensure that it is understandable to adolescents, and measures beliefs about thought processes, with particular emphasis on intrusive thoughts and concerns. It comprises five sub-scales: (1) positive beliefs about concerns; (2) negative beliefs about lack of control and concern risk; (3) cognitive confidence; (4) the need to control thoughts; and 5) cognitive self-awareness. Like MCQ- 30, answers come in a scale ranging from 1 (I disagree) to 4 (I strongly agree). Therefore, the range of scores is 30–120 in total and 6–30 in each subscale. Higher scores denote confidence in metacognitive beliefs. The study yielded a Cronbach’s αs of 0.86, (subscales αs range 0.72–0.93).

#### STAI Test Anxiety Inventory (Spielberger, 1982)

It comprises 20 items that measure anxiety. It comprises two subscales: (1) concern; and (2) emotionality. The answers come in a scale ranging from 1 (almost never) to 4 (nearly always). The range of scores is 8–32. High scores denote anxiety. The STAI’s has excellent internal consistency (αs from 0.90 to 0.91), a consistent factorial structure, and convergent validity with other anxiety measures (Spielberger, 1980).

#### MWQ questionnaire (Mrazek et al., 2013)

5-item questionnaire that measures wandering mind. Answers come in a 6-point Likert scale ranging from 1 (almost never) to 6 (nearly always). Questions include “I have difficulty maintaining focus on simple repetitive work”, and “I do things without paying full attention”. The total MWQ score is the aggregate of all elements, ranging 5–30. The Spanish version was used (Salavera et al., 2017), with a Cronbach α coefficient of 0.91.

#### ERQ-CA Emotional regulation adolescent’s questionnaire (Gullone & Taffe, 2012)

It assesses emotions and the way they are handled or regulated. It comprises 10 items divided into two subscales: (1) cognitive re-evaluation, with 6 items that measure the regulation of emotions when they emerge, changing emotional experience; and (2) expressive suppression, with 4 items that measure how emotions are expressed in order to conceal the lived experience, but without altering it. The Spanish version (Pastor et al., 2019) was used, with a Cronbach α coefficient of 0.83 in the cognitive re-evaluation scale and 0.75 in the suppression scale.

### Protocol

The sample was selected in cooperation with local schools. Parents and guardians of participants signed an informed consent form. This research consisted of two studies, which is necessary for addressing the validation of a psychological instrument and ensuring its reliability, validity, and usefulness (Hawkins et al., 2019). In this way, both exploratory validation (identifying the factors and testing the initial structure of the instrument) and confirmatory validation (confirming the structure identified in the first study with a different sample) can be carried out (López & González, 2023). This approach helps improve the reliability and validity of the instrument, as well as prevent biases and ensure its practical applicability. In both studies, the questionnaires were individually completed in the classroom, and one of the researchers was always at hand to address questions. Participation was volunteer and anonymous. Stress was put on the sincerity of the answers, and it was emphasised that the answers had no effect on school marks. Prior to the study, participants were explained the objective of the study, and the importance of answering all questions was underlined. Participants were given 45 min to complete the questionnaires. The study was designed as a lateral comparative with natural groups study, with groups formed by stable independent variables: The groups belonged to the same culture, allowing for individual comparisons and the consideration of both dependent and independent variables (Ato & Vallejo, 2015). The data was collected in January and February 2023.

### Data analysis

#### Study 1

Descriptive statistical analysis was undertaken to stablish the sociodemographic characteristics of the data: means and standard deviations for quantitative variables and percentages for nominal variables. Reliability and validity were calculated to establish the psychometric characteristics of the questionnaire. For reliability, Cronbach’s α was used to measure internal consistency, including that of scales and subscales. For convergent validity, the Pearson’s correlation coefficient of each element and subscales was calculated, to establish whether there were correlations above 0.40 and thus potential pairwise bidirectional lineal correlations between continuous variables. In addition, correlations between partial scales and aggregated scales and subscales were calculated. Factor analysis, with initial factor extraction, using main component Oblimin rotation, was undertaken to establish its validity. Oblimin rotation was chosen because it allows for the possibility of correlations between factors, which is often more realistic in psychological constructs where different dimensions may not be entirely independent. This approach enhances the interpretability of the factors and aligns with the theoretical assumptions underlying the constructs being measured. This method is widely used to design questionnaires. For the inclusion of elements in factors, factor weights of 0.400 or more were considered. The Kaiser–Meyer–Olkin’s index and Bartlett’s sphericity test were undertaken to establish the validity of the factor model.

#### Study 2

Given that the intention was to analyse the consistency of the factors of the scale, in this second study, and using the results from the exploratory factor analysis (EFA) as a reference, a confirmatory factor analysis (CFA) was conducted. The idea was that the combined use of EFA and CFA allows for more consistent results in the psychometric indices of new scales (Morgado et al., 2018). This method, which considers the combined use of EFA and CFA during the evaluation of construct validity for new measures with the goal of providing more consistent psychometric results, has recently been refuted (Abad et al., 2024). Additionally, confirmatory analyses were conducted using the AMOS program, version 26.0, with the study sample to verify whether the factorial structure of the Spanish version and the original version matched. To do this, the maximum likelihood estimation method was used instead of the weighted least squares method, due to the small sample size and the limited number of variables involved, following the recommendations of Batista and Coenders (2000). Since the variables were measured at the ordinal level, estimates were made using polychoric correlation matrices instead of covariance matrices.

The following goodness of fit indicators were calculated: chi-square (*χ*^*2*^); comparative fit index (CFI); Tucker-Lewis index (TLI); and Root Mean Square Error of Approximation (RMSEA) with a confidence interval of 90%. Based on typical criteria, CFI and TLI scores of 0.90 and 0.95, respectively, are regarded as good and excellent fit, while RMSEA values below 0.08 and 0.06 are regarded as acceptable and excellent fit, respectively. Previously, an exploratory factor analysis (EFA) was undertaken. Extraction used the maximum likelihood with varimax rotation method. The Kaiser–Meyer–Olkin index and Bartlet’s sphericity test were also used. For the extraction of the number of factors the self-value criterion above 1 was used, and to assign items to factors, factorial weights above 0.40 were considered. The psychometric characteristics of the MCQ-A questionnaire were calculated by confirmatory factor analysis (AFC) with five latent factors.

Finally, weighted undirected network analysis was undertaken with software JASP, v. 0.10.2 (JASP Team, 2019) to assess and explore structural dynamics among reactive, using the Fruchterman-Reingold algorithm (Fruchterman & Reingold, 1991). The graphs include nodes that represent variable indicators, and edges (lines) that represent relationships between nodes (Hevey, 2018). Similarly, centrality indices were calculated to estimate the interconnection of the network’s structure; the most central indicator has a stronger influence on the other features of the network (Epskamp & Fried, 2018). This network analysis allows for the visualisation of complex relationships and helps to understand how different variables interact within the questionnaire. Examining the network structure helps identify key nodes that may be playing a fundamental role within the questionnaire. Additionally, the strength and directionality of the connections between nodes allow us to observe which variables are related and how they are related. The use of this approach enables us to understand the interactions between variables beyond simple correlation or causality models, which is useful in this case.

## Results

Since the objective of the study was to assess the translated MCQ-A questionnaire in adolescents, descriptive statistics were performed (Table 1) to establish the translated version’s (Table 2). This analysis is informative about the number of elements (variables) included in the analysis and the value of Cronbach’s α reliability coefficient. Values above 0.8 can be regarded as good (the actual value was 0.85), so the scale is regarded as internally consistent.

**Table 1:** Descriptive statistics of the sample

	Men		Women		Total		*Cohen’s d*
	*M*	sd	*M*	sd	*M*	sd	Effect
Positive beliefs	11.81	3.86	11.14	3.26	11.29	3.41	.19
Negative beliefs	11.16	3.66	12.36	3.75	12.10	3.76	-.31
Cognitive confidence	9.67	3.11	10.37	3.87	10.21	3.72	-.18
Need to control thoughts	12.53	3.50	12.18	3.58	12.25	3.56	.09
Cognitive self-knowledge	16.55	3.61	16.08	3.44	16.18	3.48	.13
Wandering mind	14.85	3.76	13.47	4.47	13.77	4.36	.31
Feature-anxiety	38.46	8.22	43.93	10.40	42.72	10.20	-.53
Cognitive re-evaluation	15.53	3.16	16.38	3.79	16.19	3.67	-.23
Expressive suppression	13.63	3.70	13.11	3.48	13.22	3.53	.14

**Table 2:** Internal consistency MCQ-A

Items	Mean	SD	α if suppressed	Scale
1	2.21	.81	.85	
2	2.08	.84	.85	
3	2.96	.85	.84	
4	1.73	.89	.85	
5	2.87	.76	.86	
6	2.08	.88	.85	
7	1.64	.84	.85	
8	1.72	.94	.85	
9	2.14	.99	.84	
10	1.91	.81	.85	
11	2.47	.99	.85	
12	2.60	.87	.85	
13	1.92	.86	.84	
14	2.09	.77	.85	ω =.85
15	1.50	.77	.84	α =.85
16	2.83	.71	.85	
17	1.70	.87	.85	
18	2.68	.83	.85	
19	1.99	.84	.85	
20	1.62	.80	.85	
21	2.18	.96	.84	
22	2.08	.93	.84	
23	1.95	.75	.85	
24	1.64	.89	.85	
25	2.48	.99	.85	
26	1.51	.75	.85	
27	2.06	.87	.84	
28	1.55	.72	.85	
29	1.53	.76	.85	
30	2.18	.88	.84	

This was followed by scale factor analysis (Table 3). In order to enable comparisons, it was decided to regard that the model was a good fit to the data if the function between Chi-square and degrees of freedom was not above 3 (Hu & Bentler, 1999). In this study, the scales yielded results below 3, suggesting a good fit and internal validity.

**Table 3:** Exploratory factor analysis

	Factors				
	1	2	3	4	5
1			.616		
2					.520
3		.612			
4					.708
5		.658			
6	.617				
7			.691		
8				.808	
9					.802
10			.789		
11					.622
12		.706			
13	.539				
14				.520	
15					.628
16		.759			
17				.799	
18		.750			
19			.741		
20	.586				
21					.724
22	.634				
23			.724		
24				.633	
25	.542				
26				.858	
27	.706				
28			.639		
29				.785	
30		.650			
Alfa Cronbach subscale	.741	.797	.801	.831	.769
Eigenvalues	6.300	3.521	2.644	2.143	1.418
Explained variance	21.001	11.738	8.813	7.145	4.726
Aggregate explained variance	21.001	32.739	41.552	48.697	53.422

*Extraction method* main component analysis, *Rotation method* Oblimin with Kaiser normalisation

Factorisation was undertaken with main component analysis with Varimax rotation, once the viability of the factor analysis was confirmed under the following criteria: the relational matrix presents a large number of correlations (86.9%) with a value above 0.30 and determinant equal to 0.002. The result of Bartlett’s sphericity test demonstrated that the variables were not independent (Bartlett test = 2607.08, *p* < 0.001). The result of the Kaiser Meyer Adequacy (KMO) test was 0.822, indicating that pairwise correlation of variables can almost be explained by the remaining variables. All the Measures of Sampling Adequacy (MSA) values were above 0.83. These values endorse the factor analysis of the correlational matrix. As shown in Table 1, five factors with eigenvalue above 1 were obtained, taking into account factors with a factor weight above 0.40, which explains 53.42% of total variance.

**Fig. 1:**
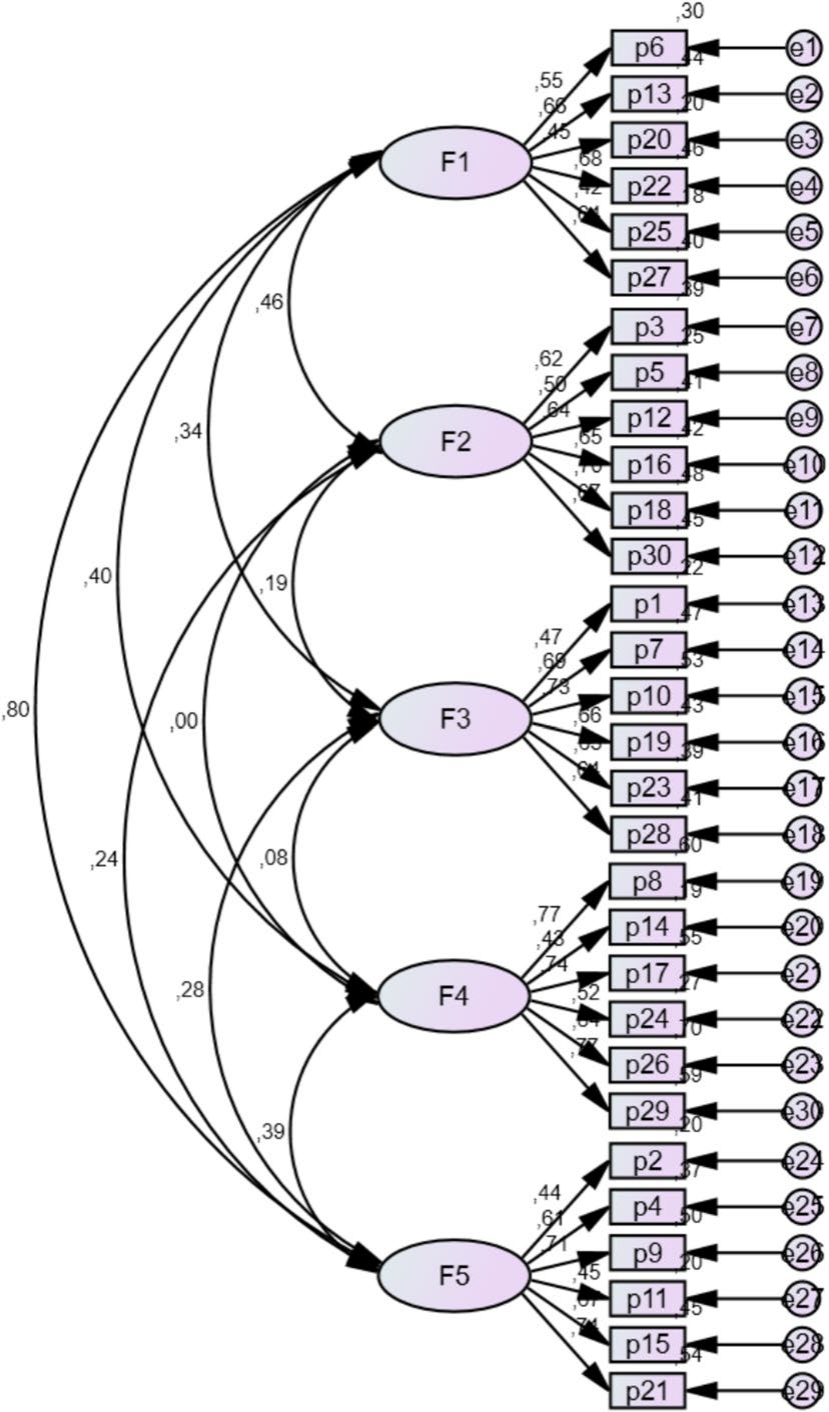
Estimated normalised parameters of the CFA model

Confirmatory factor analysis (CFA) was used to establish if the five-factor structure of the MCQ-A fit the data. Figure 1 shows the CFA results for the model generated in the exploratory study, with structural variations, using the maximum likelihood extraction method. This confirmed the fitness of the model, as the result was a sustainable model which comprises the five factors identified and 30 indicators in total. Standard regression coefficients were statistically significant (*p* < 0.001) with values above 0.4, which suggests that all indicators are satisfactorily saturated with their respective latent variables. Factor covariance was − 0.62 (not above 0.8), ruling out collinearity issues and suggesting its discriminating value. Concerning the fitness of the model, the various fitness indexes yielded good results, so it can be argued that the model suggested for the factor structure is sustainable: *χ*^*2*^(395) = 849.497, *p* < 0.001; *χ*^2^/gl = 2.151; GFI = 0.92; CFI = 0.96; NFI = 0.93; TLI = 0.92; RMSEA = 0.08, IC 95% (0.02–0.14).

Concerning convergent validity (Table 4), it was examined if the MCQ-A scale was in any way related to the other scales (Feature-Anxiety; Emotional Regulation; Wandering Mind). The results yield correlations between the MCQ-A and the wandering mind and feature-anxiety questionnaires, but not with emotional regulation scales (cognitive re-evaluation and expressive suppression).

**Table 4:** Convergent validity

	1	2	3	4
1. Metacognition MCQ-A				
2. Wandering mind	.323**			
3. Feature-anxiety	.603**	.422**		
4. Cognitive reevaluation	.068	.005	.523	
5. Expressive suppression	.052	.101	.112	.582**

^*^p < 0.05 **p < 0.01

**Fig. 2:**
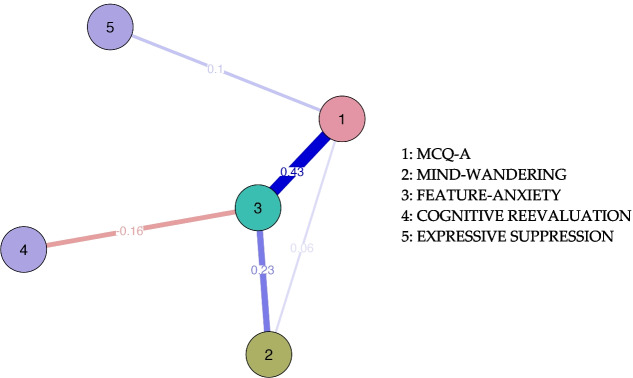
Network analysis of constructs. Note: Each line (edge) represents partial correlations between two variables; thickness represents the strength of the correlation

Finally, network analysis was undertaken, as illustrated in Fig. 2. In network analysis, it is represented by nodes and edges. Each node represents a variable being studied, and the edges are the connections or relationships between these nodes, representing how the elements of the network interact with each other. In the present study, six nodes and fifteen edges different to zero are observed, connecting the nodes. Basically, correlations in network analysis can be positive, when both variables increase together; or negative, when one increases and the other decreases; and can present strong or weak correlations, indicating the intensity of that relationship, which allows for inferences about the behavior and structure of the system.

The analysis represents positive correlations (blue edges) that denote vital connections. Correlations were stronger between nodes 1 MCQ-A and 3 (feature-anxiety) (partial *r* = 0.43, *p* < 0.01) and nodes 2 (wandering mind) and 3 (feature-anxiety) (partial *r* = 0.23, *p* < 0.01). Nodes 3 (feature-anxiety) and 4 (negative re-evaluation) (parcial *r* = − 0.16, *p* < 0.01) are negatively correlated, which means the higher scores in one implies lower scores in the other.

**Fig. 3:**
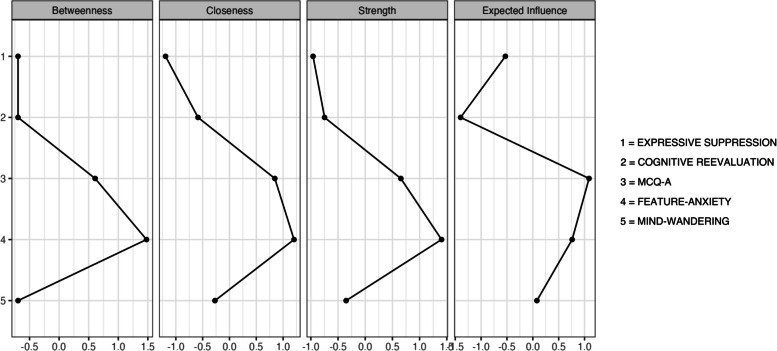
Centrality graph

Figure 3 represents the measure of strength, owing to the greatest stability of this variable in network models (Bringmann et al., 2019). The measure of strength in network analysis is a way to quantify the intensity of relationships between variables in a network. A high strength indicates that a variable has a significant and stable impact on others, making it a key factor in the network's dynamics. In the present study, the highest value was yielded by reactive 4 (feature-anxiety).

Unlike emotional regulation (cognitive re-evaluation and expressive suppression), mind-wandering and feature-anxiety were found to be positive predictors of MCQ-A (Table 5).

**Table 5:** MCQ-A dimension predictors

	Beta	*t*
(Constant)	34.446	21.840**
Mind-Wandering	.214	2.457**
Feature-anxiety	.581	15.573**
Cognitive re-evaluation	.049	1.529
Expressive suppression	−.044	− 1.343
*Adjusted R*^*2*^ (%)	58.6	
Model	*F* = 170.617; *p* < 0.001	

^**^p < 0.01

## Discussion

The aim of this work was to establish the utility of the MCQ-A questionnaire to measure non-adaptive metacognitive beliefs in adolescents, as well as to undertake a confirmatory factor analysis of the scale. For this, the factor structure of MCQ-A and the concurrent associations of metacognitive beliefs and other related constructs, such as feature-anxiety, wandering mind, and emotional regulation (cognitive re-evaluation and expressive suppression), had to be established.

This study validates the adapted and translated MCQ-A by assessing its psychometric characteristics. The method used to establish the cultural and linguistic equivalence of the original and the adapted scales is presented, including analyses of internal consistency and validity of factor structure. Factor analysis confirmed the validity of the five-factor model for both adults and adolescents. The MCQ-A presents a five-dimension structure with highly-saturated items, which reflects high internal consistency (0.85), similar to that of the original scale used with adults (Cartwright & Wells, 1997). All items were weighted in their respective subscales (α between 0.84 and 0.85). The results clearly showed that the components of the MCQ-A questionnaire present an aggregate variance of 53.42%. Additional evidence is presented for validity in relation to other variables, as it was shown that the aggregate of the components of the MCQ-A is reliable in the different subscales, replicating reliability and validity results of previous studies with adult populations (Cook et al., 2014; Fisher et al., 2016; Grøtte et al., 2016; Spada et al., 2016). After exploring the underlying factor structure of the metacognitive beliefs questionnaire, it was observed that the reliability indexes in our study and the original scale are similar in the five subscales. It can thus be argued that the translated and adolescent-adapted version maintains the scale’s original consistency. This empirically validates the five-component structure.

The five-component MCQ-A questionnaire fully agrees with Cartwright & Wells’s (1997) original proposal. The adapted questionnaire was a good fit for the data, according to the five-component confirmatory factor analysis. The data indicates that it is a good tool to establish non-adaptive metacognitive beliefs in adolescents. The overall results confirm the robustness and utility of the MCQ-A to measure metacognitive beliefs.

The main limitation of this study is its lateral nature; longitudinal studies are necessary to track the evolution of metacognitive beliefs. In addition, positive psychologists are often accused of focusing exclusively on developed western countries, so future studies should include other countries and cultures (Selin & Davey, 2012). Research can, and must, also consider other constructs, including social skills, problem resolution, and self-esteem, and establish their relationship with these variables and improve our understanding of metacognitive knowledge (Shea & Frith, 2019).

For the future, although much works remains to be done concerning the assessment of metacognitive beliefs, our results clearly establish the importance of addressing these issues early on. Specific educational programmes must be implemented, as metacognitive knowledge has the potential to improve academic, personal, and emotional development. The results of this study encourage us to continue asking new questions and seeking new answers to move forward in the socio-emotional development of the person.

## Conclusion

The main conclusion of the study is that the MCQ-A questionnaire allows the assessment of metacognitive beliefs in Spanish adolescents, proving to be a consistent, valid, and easy-to-administer psychometric tool. The study has confirmed the validity of the five-factor factorial structure. Additionally, its concurrent validity has been assessed in relation to anxiety and emotional regulation values in this population. This work will contribute to a more accurate measurement of metacognition in Spanish adolescents and may help teachers in their classroom work, enabling the use of clearer educational guidelines. Finally, the implications of these findings should not be underestimated, and prioritizing work on this idea should become a key focus for all professionals working with adolescents.

## Data Availability

The data presented in this study are available upon request from the corresponding author.

## Abbreviations

MCQ-A: Metacognition Questionnaire-Adolescents
CFI: Comparative fit index
TLI: Tucker-Lewis index
RMSEA: Root mean square error of approximation
EFA: Exploratory factor analysis
AFC: Confirmatory factor analysis
